# Autism spectrum disorder burden across 798 locations, 1990–2023: frontier and inequality mapping in the context of child injury prevention and safety promotion

**DOI:** 10.3389/fpubh.2026.1930391

**Published:** 2026-09-09

**Authors:** Xuezhi Liang, Wenyi Jin, Xinyan Bu, Minglu Yuan, Meng Zheng, Shiyu Feng, Liling Zhu, Yutong Lu, Jing-Xuan Zhou, Nadia M. Hamdy, Claire Chenwen Zhong, Queran Lin, Xiaoliang Huang, Wen Chen, on behalf of GBD Child Autism Injury Collaborators

**Affiliations:** 1 Department of Medical Statistics, School of Public Health, Sun Yat-sen University, Guangzhou, China; 2 Sun Yat-sen Center for Migrant Health Policy, Sun Yat-sen University, Guangzhou, China; 3 Department of Orthopaedics, Renmin Hospital of Wuhan University, Wuhan University, Wuhan, China; 4 Department of Biomedical Sciences, City University of Hong Kong, Hong Kong, Hong Kong SAR, China; 5 Guangdong Provincial Key Laboratory of Malignant Tumor Epigenetics and Gene Regulation, Breast Tumor Center, Sun Yat-sen Memorial Hospital, Sun Yat-sen University, Guangzhou, China; 6 National Supercomputer Center in Guangzhou, Sun Yat-sen University, Guangzhou, China; 7 School of Urban Planning and Design, Peking University Shenzhen Graduate School, Shenzhen, China; 8 Department of Biochemistry, Faculty of Pharmacy, Ain Shams University, Cairo, Egypt; 9 JC School of Public Health & Primary Care, Faculty of Medicine, The Chinese University of Hong Kong, Hong Kong, Hong Kong SAR, China; 10 Guangdong-Hong Kong Joint Laboratory for RNA Medicine, Breast Tumor Center, Guangdong Provincial Clinical Research Center for Breast Diseases, Clinical Research Center, Sun Yat-sen Memorial Hospital, Sun Yat-sen University, Guangzhou, China; 11 WHO Collaborating Centre for Public Health Education and Training, Department of Primary Care and Public Health, School of Public Health, Faculty of Medicine, Imperial College London, London, United Kingdom; 12 Government Affairs Service Centre of Health Commission of Guangdong Province, Guangzhou, China

**Keywords:** autism Spectrum disorder, child injury prevention, health inequality, safety promotion, subnational analysis

## Abstract

**Introduction:**

Autism spectrum disorder (ASD) is commonly identified in childhood and can affect communication, adaptive functioning, supervision, and access to health and educational services. These needs may be relevant to child safety planning, but this study did not measure injury outcomes.

**Methods:**

We analyzed Global Burden of Disease 1990–2023 estimates of ASD incidence and disability-adjusted life-year (DALY) rates across 798 locations. Age-standardized and under-20 rates were benchmarked against a Socio-demographic Index-related frontier using free disposal hull analysis. Socioeconomic inequalities were assessed using the slope index of inequality and concentration index.

**Results:**

In 2023, 98 countries and territories (48.0%) were classified as behind the age-standardized incidence frontier and 78 (38.2%) as behind the age-standardized DALY frontier. Among individuals younger than 20 years, 100 (49.0%) were behind the incidence frontier and 123 (60.2%) were behind the DALY frontier. Frontier deviation increased in most countries between 1990 and 2023. Sweden had the largest reduction across all four national metrics, whereas Japan had the largest increase. Japan also had the largest 2023 deviations for age-standardized incidence, age-standardized DALYs, and under-20 DALYs; Brunei Darussalam had the largest under-20 incidence deviation. Selected Japanese prefectures were prominent subnational outliers, while locations in Pakistan and Nigeria showed the largest reductions. Recorded under-20 DALY burden became more concentrated in higher-SDI populations globally, whereas disadvantaged populations within low-SDI settings retained substantial absolute inequality.

**Discussion:**

These findings provide descriptive benchmarks for ASD surveillance, early recognition, service organization, and equitable healthcare planning. They should not be interpreted as direct measures of service quality, unmet need, or injury risk.

## 1 Introduction

Autism Spectrum Disorder (ASD) is a lifelong neurodevelopmental condition characterized by difficulties in social communication, repetitive behaviors, sensory sensitivities, and frequent co-occurring intellectual disabilities of varying severity (1–3). ASD poses a substantial global public health challenge, with its impacts spanning health, education, and broader societal domains (4, 5). From a child injury-prevention perspective, delayed recognition matters because wandering, communication barriers, and caregiver strain and so on, can change exposure to preventable harms such as drowning, ingestion events, burns, traffic-related injury, and self-harm (6–10). Although ASD affects individuals across the life course, its first major service gateway is usually in childhood, when families seek developmental assessment, early intervention, school support, and practical guidance for everyday safety. These child-facing needs are especially difficult to meet when recognition is delayed, care pathways are fragmented, specialist capacity is uneven, or families face social and economic barriers.

Most ASD service pathways begin in childhood. Families usually enter the system through developmental concerns, diagnostic assessment, early intervention, school support, or requests for help with daily safety and behavior. When recognition is delayed, or when diagnostic and post-diagnostic services are fragmented, children may miss the window in which caregiver guidance, school-based planning, and practical safety support are most useful. These processes are relevant to everyday safety, but they cannot be inferred directly from population-level incidence or DALY estimates.

Existing comparative estimates show a substantial global burden of ASD, but national averages are poorly suited to identifying where services are failing children and families (1, 11–14). Frontier analysis compares recorded rates with the lowest rates observed at similar levels of development. Inequality analysis evaluates socioeconomic gradients in recorded burden. Together, these approaches provide descriptive benchmarks across geographic scales. They do not identify the mechanisms that produce a deviation. Differences may arise from diagnostic practices, case ascertainment, surveillance, data availability, population structure, or service organization.

In this study, we used 1990–2023 comparative health estimates to map ASD incidence and DALYs rates at global, regional, national, and subnational levels. We applied frontier analysis to age-standardized and under-20 incidence and DALYs rates, and inequality analysis to assess absolute and relative socioeconomic gradients in ASD burden. By emphasizing children and adolescents, this study links ASD burden estimation with child safety prevention.

## 2 Methods

We used publicly available GBD estimates for 1990–2023 generated through a standardized international disease-burden framework and accessed through the Global Health Data Exchange Platform (14, 15). Drawing from an extensive array of data sources, including national surveys, disease registries, and vital records, the study provides age-specific estimates of key health metrics for 204 countries and territories, and subnational locations, totaling 798 locations. Its scope spans global, regional, and subnational levels, offering critical insights for policymakers seeking to address and mitigate health disparities. To ensure consistency and comparability, the study employed robust statistical methodologies grounded in a standardized framework developed by the Institute for Health Metrics and Evaluation (IHME) (16). Data integration and modeling were facilitated through the Global Health Data Exchange (GHDx) platform,^1^ and additional data can be requested from Institute for Health Metrics and Evaluation. The study adhered strictly to the Guidelines for Accurate and Transparent Health Estimates Reporting (GATHER) and employed anonymization protocols, further confirm its scientific rigor and ethical integrity for global applicability (17).

We examined incidence rates and DALY rates per 100,000 population. Frontier analyses used age-standardized rates and rates among individuals younger than 20 years. Inequality analyses were conducted for all-age and under-20 rates. Age-standardized measures support comparisons across populations with different age structures, whereas age-specific and all-age measures describe the burden in the populations represented.

Frontier analysis estimated the lowest observed ASD burden at each level of SDI. We used the free disposal hull (FDH) because it is non-parametric, does not require a prespecified functional form or error distribution, and preserves observed best performance. Unlike stochastic frontier analysis, FDH does not require a distributional model; unlike conventional data envelopment analysis, it does not impose a convexity assumption (18, 19). The results were then smoothed using locally weighted polynomial regression (LOESS), which reduces noise and captures local trends (20). For each location-year, frontier deviation, termed the effective difference, was calculated as the location-specific GBD rate estimate minus the smoothed frontier rate at the same sociodemographic index (SDI) (11). Larger positive values indicated greater distance from the lowest rate observed among locations with comparable development. A location was classified as having attained the frontier when the frontier estimate lay within the 95% uncertainty interval (UI) of the location-specific GBD estimate. It was classified as behind the frontier when the location-specific estimate was higher and its 95% UI did not include the frontier estimate. Frontier deviation is a comparative epidemiological benchmark. It is not a direct measure of health-system efficiency, service quality, unmet need, or injury risk.

Inequality analysis was conducted to assess disparities in ASD burden using the Slope Index of Inequality (SII) for absolute inequality and the Concentration Index (CI) for relative inequality (21, 22). The SII measures the absolute inequality in ASD burden between the most and least socioeconomically advantaged groups, with values theoretically ranging from negative infinity to positive infinity; larger absolute values reflect greater absolute disparities. The CI quantifies relative inequality across the entire socioeconomic spectrum, ranging between −1 and +1, where values farther from zero indicate more pronounced relative inequalities. Additionally, positive values of SII and CI indicate that ASD burden concentrated among the more developed populations, while negative values indicate a higher concentration in less developed populations.

Analyses were conducted at global, SDI-regional, national, and available subnational levels. The subnational component was descriptive and aspatial. Administrative units were not modeled through adjacency matrices, spatial autocorrelation, urban–rural categories, built-environment measures, provider density, or travel-time accessibility. These indicators were not available in a harmonized form across the included locations.

To ensure robust estimation, 500 iterations were performed at each modelling step by sampling from the posterior distribution of estimates, with calculations completed separately for each iteration. From these 500 draws, a subset of 100 draws was randomly selected to calculate mean estimates for each year, age group, sex, and location. The 95% uncertainty levels (UI) were derived by ranking these 100 draws and identifying the 2.5th and 97.5th percentile values (23). Age-standardized estimates were calculated using standard population age weights in this study. All estimates are presented as rates per 100,000 population. Statistical analyses and visualizations were performed using R software (version 4.2.0) and Python (version 3.9.19).

## 3 Results

### 3.1 Global overview

**Figure 1:**
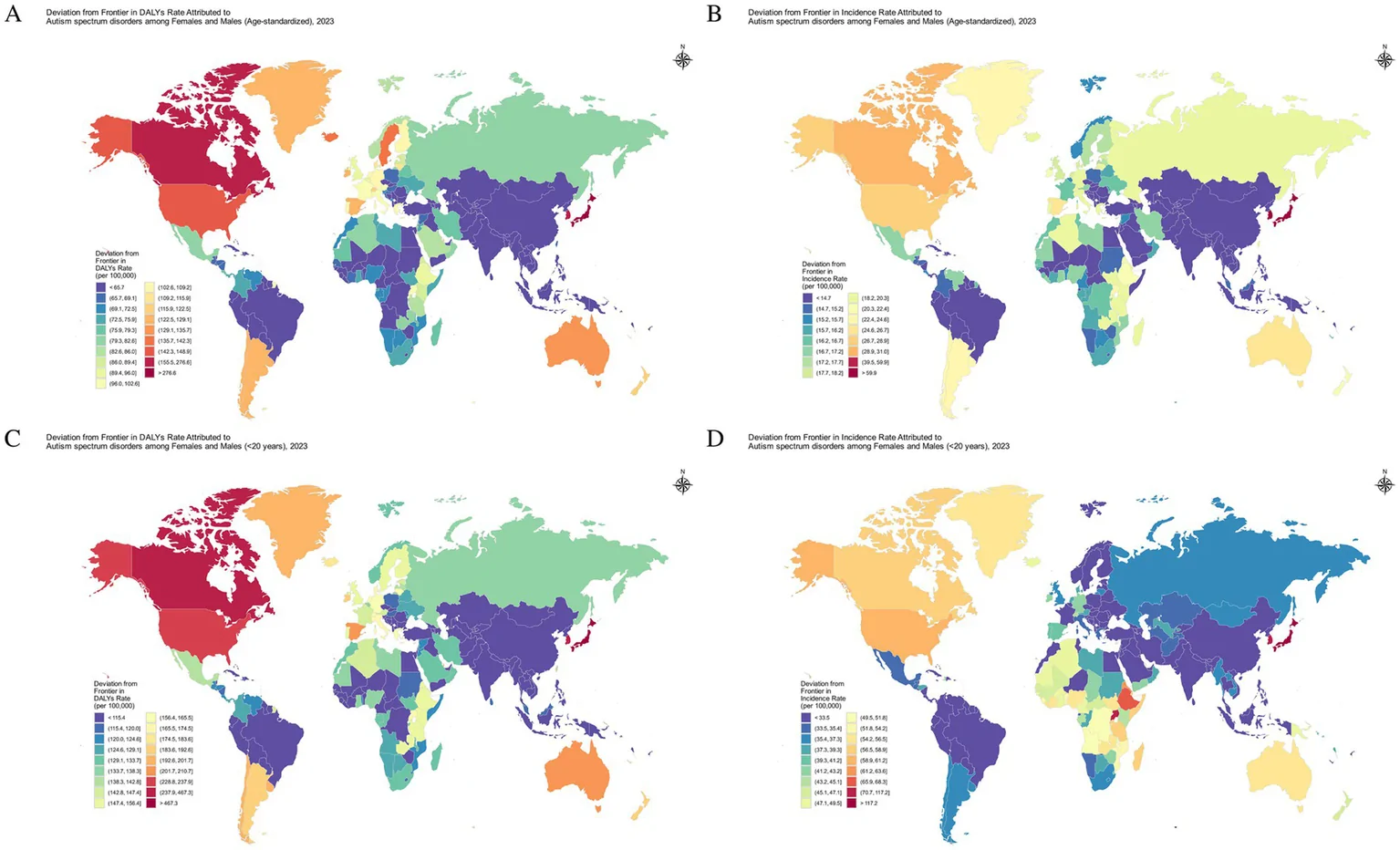
Global distribution of deviations from the estimated frontier for ASD burden in 2023. **(A)** Frontier deviation in the DALYs rate; **(B)** frontier deviation in the age-standardized incidence rate; **(C)** frontier deviation in the DALYs rate among individuals younger than 20 years; and **(D)** frontier deviation in the incidence rate among individuals younger than 20 years. Countries and territories are color-coded according to the magnitude of frontier deviation, ranging from dark blue for the smallest deviations to dark red for the largest deviations. Rates are reported per 100,000 population. ASD, autism spectrum disorder; DALYs, disability-adjusted life-years.

The 2023 analyses revealed substantial regional and inter-country variations in deviations from the frontier analysis for age-standardized DALYs rates and incidence rates of ASD, with child adolescent metrics adding a distinct layer to the age-standardized estimates. In the global maps, deviations in age-standardized and DALYs rates of less than 20 years were profound in Japan and North America, and several high-income settings, whereas many countries in South Asia, Latin America, and parts of Sub-Saharan Africa remained closer to the frontiers (Figure 1). The map for age-standardized population and individuals younger than 20 years, incidence rates showed a broadly similar high-deviation pattern in Japan.

**Figure 2:**
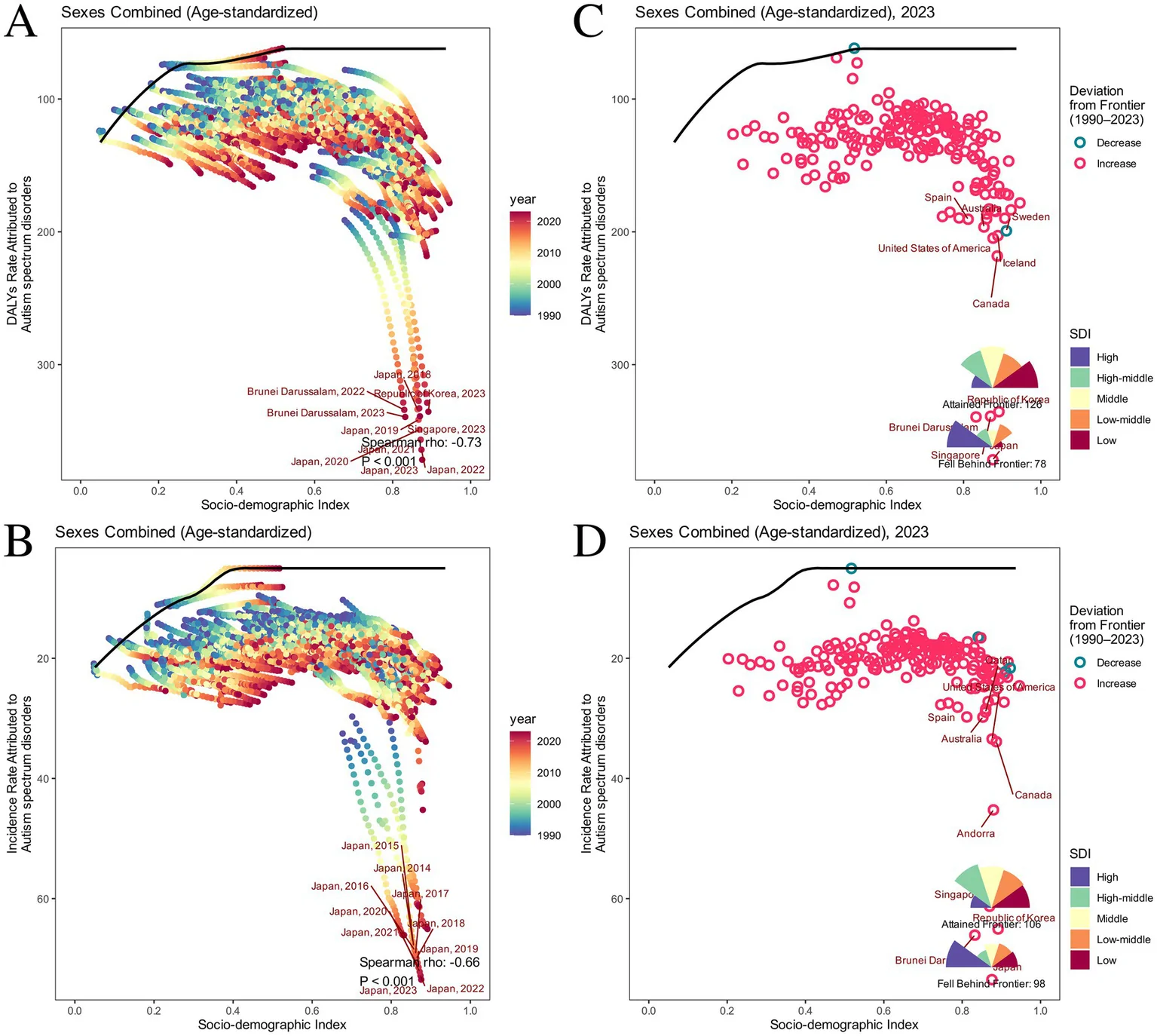
Frontier analysis of ASD burden with age-standardized metrics and sociodemographic development, 1990–2023 and 2023. **(A)** Frontier analysis between the SDI and age-standardized DALYs rate for ASD from 1990 to 2023. Each point represents a country-year, color-coded by year. The black line marks the estimated frontier, indicating the lowest burden at a given SDI level. Selected countries with notable shifts are labeled. **(B)** Frontier analysis for DALYs rate in 2023. Countries above the frontier have a higher-than-expected burden. Circle size and color suggest deviation magnitude and direction from 1990 to 2023: green indicates a decrease in ASD burden, red shows an increase trend through 30 years. **(C)** Frontier analysis between the SDI and age-standardized incidence rate for ASD from 1990 to 2023 across countries. The black line represents the frontier, capturing the lowest incidence at each SDI level. **(D)** Frontier analysis for incidence rate in 2023. Circle size and color suggest deviation magnitude and direction from 1990 to 2023: reen indicates a decrease in ASD incidence rate, red shows an increase trend through 30 years. SDI. Sociodemographic Index; DALYs, Disability-adjusted life-years; ASD, autism spectrum disorder.

At the country and territory level, 78 countries and territories (38.2%) fell behind the age-standardized DALYs frontier, and 98 (48.0%) fell behind the age-standardized incidence frontier in 2023 (Table 1). Among individuals younger than 20 years, 123 countries and territories (60.2%) were behind the DALYs frontier, and 100 (49.0%) were behind the incidence frontier (Table 1; Figure 2). Detailed results across the 204 countries and territories are presented in the Supplementary Information.

**Table 1:** Summary of frontier analysis for autism spectrum disorder burden: age-standardized incidence and DALYs rates by country.

Metric	Key findings	Age-standardized rates	<20 years
Incidence	Countries behind frontier *n* (%) in 2023	98 (48.0%)	100 (49.0%)
	Countries with largest deviation in 2023	Japan	Brunei Darussalam
	Number of countries with increasing deviation from frontiers *n* (%)	200 (98.0%)	157 (77.0%)
	Country with largest decrease in deviation (1990–2023)	Sweden	Sweden
	Country with largest increase in deviation (1990–2023)	Japan	Japan
DALYs	Countries behind frontier n (%) in 2023	78 (38.2%)	123 (60.2%)
	Countries with largest deviation in 2023	Japan	Japan
	Number of countries with increasing deviation from frontiers *n* (%)	202 (99.0%)	201 (98.5%)
	Country with largest decrease in deviation (1990–2023)	Sweden	Sweden
	Country with largest increase in deviation (1990–2023)	Japan	Japan

DALYs, Disability-Adjusted Life Years. “Frontier” refers to optimal achievable burden based on SDI by theory. Rates are reported per 100,000 population.

**Figure 3:**
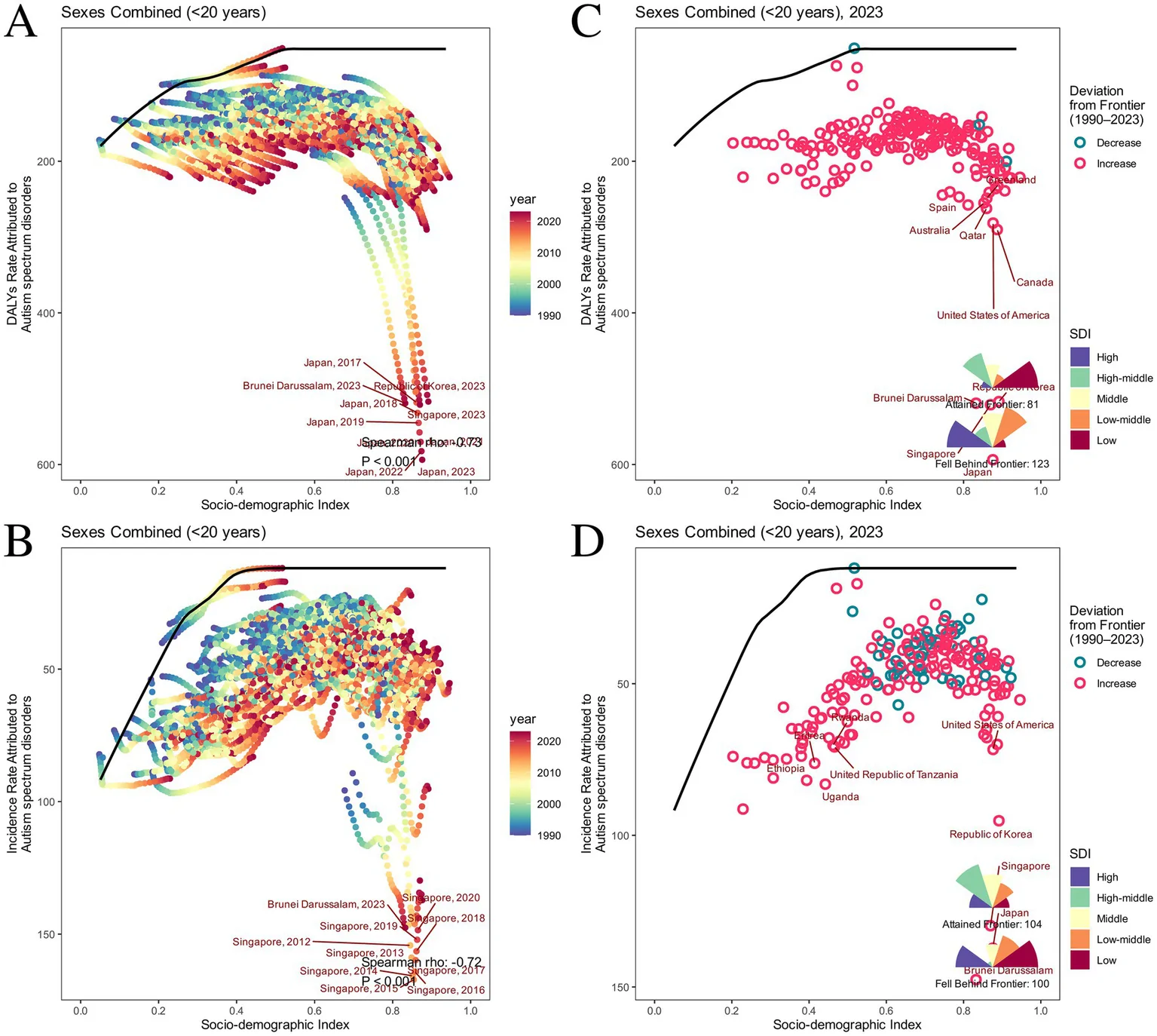
Frontier analysis of ASD burden with children and adolescents’ (under-20) metrics and sociodemographic development, 1990–2023 and 2023. **(A)** Frontier analysis between the SDI and DALYs rate of children and adolescents with ASD from 1990 to 2023. Each point represents a country-year, color-coded by year. The black line marks the estimated frontier, indicating the lowest burden at a given SDI level. Selected countries with notable shifts are labeled. **(B)** Frontier analysis for DALYs rate of children and adolescents with ASD in 2023. Countries above the frontier have a higher-than-expected burden. Circle size and color suggest deviation magnitude and direction from 1990 to 2023: Green indicates a decrease in ASD burden, red shows an increase trend through 30 years. **(C)** Frontier analysis between the SDI and age-standardized incidence rate of children and adolescents with ASD from 1990 to 2023 across countries. The black line represents the frontier, capturing the lowest incidence at each SDI level. **(D)** Frontier analysis for incidence rate of children and adolescents with ASD in 2023. Circle size and color suggest deviation magnitude and direction from 1990 to 2023: Green indicates a decrease in ASD incidence rate, red shows an increase trend through 30 years. SDI, Socio-demographic Index; DALYs, Disability-adjusted Life-years; ASD, Autism Spectrum Disorder.

From 1990 to 2023, frontier increased in 202 countries and territories (99.0%) for age-standardized DALYs, 200 (98.0%) for age-standardized incidence, 201 (98.5%) for DALYs among individuals younger than 20 years, and 157 (77.0%) for incidence among individuals younger than 20 years (Figure 2). Sweden showed the largest reduction in frontier deviation across all four national metrics, whereas Japan had the largest increase. Metric-specific changes in frontier deviation are shown in Figures 2, 3.

In 2023, Japan had the largest deviation for the age-standardized incidence, age-standardized DALYs, and DALYs among individuals younger than 20 years, with effective differences of 185.2, 309.5, and 542.0, respectively (Table 1; Figures 2, 3). Brunei Darussalam had the largest under-20 incidence deviation. Other high-deviation countries labeled in the frontier plots included the Republic of Korea, Singapore, Canada, the United States of America, Iceland, and Spain, depending on the metrics and age groups.

**Figure 4:**
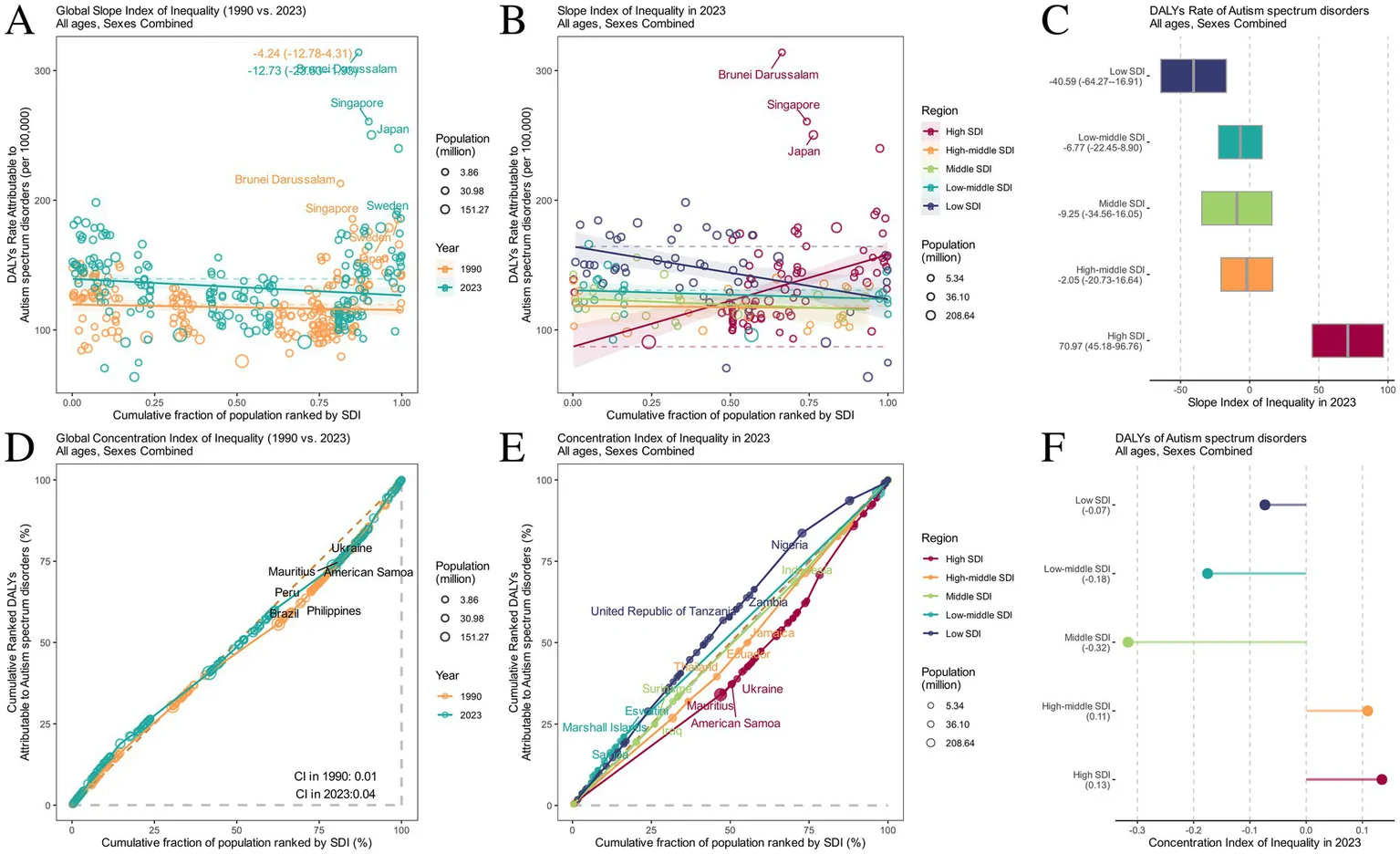
Global and regional inequality analysis of ASD all ages DALYs rate using slope index of inequality **(A–C)** and concentration index **(D–F)**, 1990–2023 and 2023. **(A–C)** Illustrate absolute inequality (slope index of inequality), which measures disparities in age-standardized DALYs rate across SDI spectrum. Trends over time and variations across SDI regions are presented. Higher absolute values indicate greater inequality in ASD burden. **(D–F)** Demonstrate the relative inequality (concentration index of inequality), which quantifies how ASD burden is distributed relative to SDI rankings. ASD, autism spectrum disorder; SDI, Socio-demographic Index; DALYs, isability-adjusted life-years.

Inequality patterns differed between all-age DALYs and DALYs among individuals younger than 20 years. For all-age DALYs rates, the global SII shifted from −4.24 in 1990 to −12.73 in 2023, while the CI increased from 0.01 to 0.04 (Figure 4). In 2023, the low SDI region showed a negative SII [−40.59 (95% UI: −64.27 to −16.91)], whereas the high SDI region showed a strongly positive SII [70.97 (45.18 to 96.76)]. The low-middle, middle, and high-middle SDI regions had smaller or more uncertain absolute gradients. These estimates show opposite socioeconomic gradients within low and high SDI strata.

**Figure 5:**
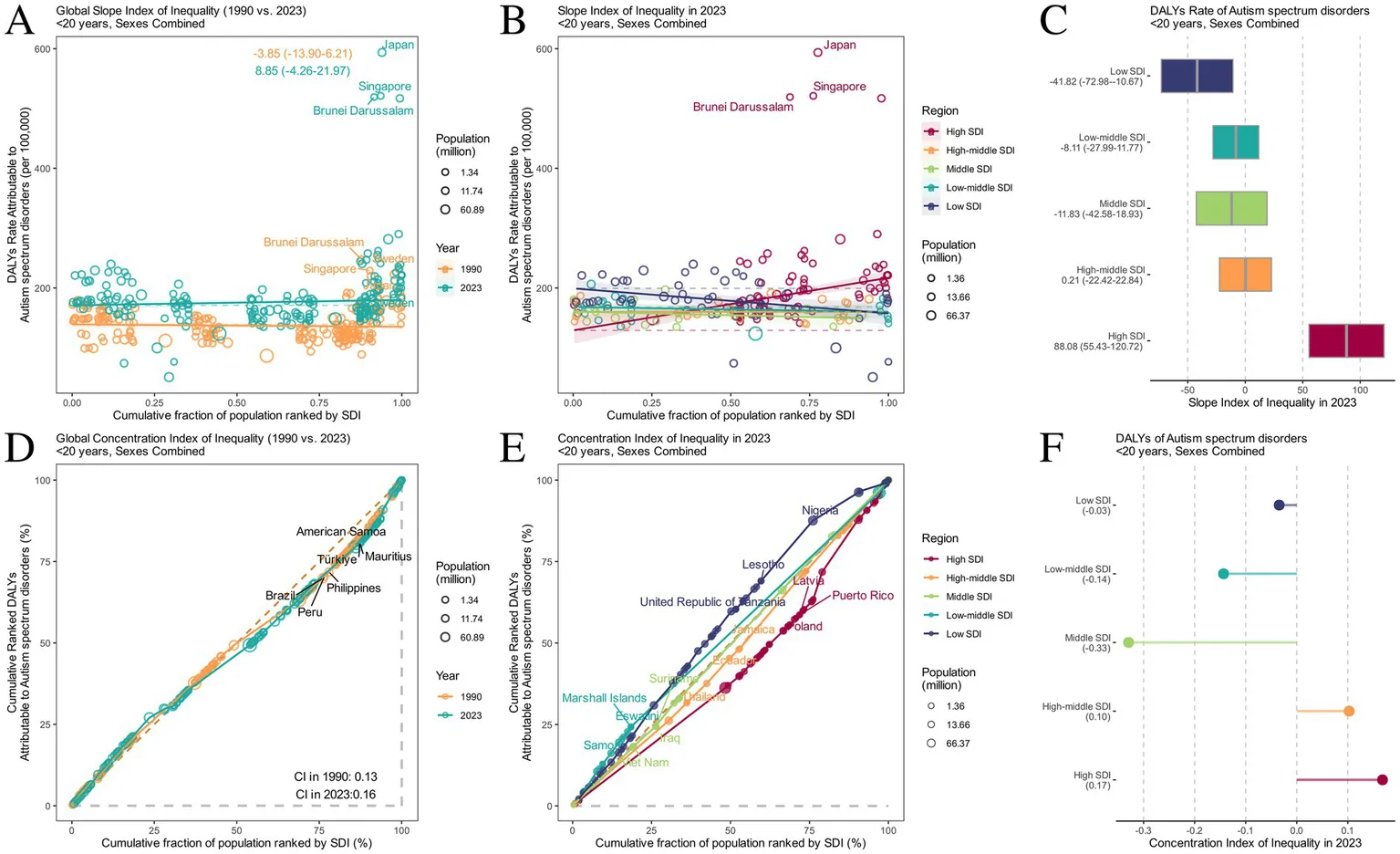
Global and regional inequality analysis of children and adolescents’ DALYs rate using slope index of inequality **(A–C)** and concentration index **(D–F)**, 1990–2023 and 2023. **(A–C)** Illustrate absolute inequality (slope index of inequality), which measures disparities in DALYs rate of children and adolescents across SDI spectrum. Trends over time and variations across SDI regions are presented. Higher absolute values indicate greater inequality in ASD burden. **(D–F)** Demonstrate the relative inequality (concentration index of inequality), which quantifies how ASD burden is distributed relative to SDI rankings. ASD, autism spectrum disorder; SDI, Socio-demographic Index; DALYs, Disability-adjusted life-years.

For children and adolescents DALYs, the global SII shifted from −3.85 (−13.90 to 6.21) in 1990 to 8.85 (−4.26 to 21.97) in 2023, while the CI increased from 0.13 to 0.16 (Figure 5). In 2023, the high SDI region showed the strongest positive absolute inequality for <20 children and adolescents’ DALYs rates [SII: 88.08 (55.43 to 120.72)], whereas the low SDI region showed a negative gradient [−41.82 (−72.98 to −10.67)] (Figure 5). The low-middle SDI, middle SDI, and high-middle SDI regions had smaller or uncertain gradients. DALYs among children and adolescents showed a global shift toward higher-SDI populations, while disadvantaged populations within low SDI settings continued to show substantial absolute inequality.

Incidence inequality among children and adolescents showed a related but distinct pattern in the supplementary analyses. The global SII remained negative in 2023 [−22.39 (95% UI: −27.09 to −17.70)], whereas the CI increased from 0.21 in 1990 to 0.25 in 2023 (Supplementary Figure S1). In 2023, the low SDI region had a greatest negative SII for incidence [−37.40 (−47.61 to −27.19)], whereas the high SDI region had the largest positive SII [21.32 (11.62 to 31.02)].

### 3.2 Subnational analysis

Subnational analyses showed fewer locations classified as behind the incidence frontier than the DALY frontier in 2023 (Table 2). For age-standardized incidence, 5 subnational locations (1.2%) fell behind the frontier, while 385 (99.0%) showed increasing deviation from 1990 to 2023. For incidence among individuals younger than 20 years, 6 locations (1.5%) fell behind the frontier, while 352 (90.5%) showed increasing deviation. For DALYs, 48 locations (12.3%) fell behind the age-standardized frontier and 48 (12.3%) fell behind the <20 years frontier; increasing deviation was observed in 384 locations (98.7%) for age-standardized DALYs and 383 locations (98.75%) for <20 DALYs (Table 2).

**Table 2:** Summary of frontier analysis for autism spectrum disorder burden: age-standardized incidence and DALYs rates by subnational locations.

Metric	Key findings	Age-standardized rates	<20 years
Incidence	Locations behind frontier *n* (%) in 2023	5 (1.2%)	6 (1.5%)
	Locations with largest deviation in 2023	Japan: Aomori	Japan: Fukuoka
	Number of subnational locations with increasing deviation from frontiers *n* (%)	385 (99.0%)	352 (90.5%)
	Locations with largest decrease in deviation (1990–2023)	Pakistan: Sindh	Nigeria: Akwa Ibom
	Locations with largest increase in deviation (1990–2023)	Japan: Aomori	Japan: Aichi
DALYs	Locations behind frontier *n* (%) in 2023	48 (12.3%)	48 (12.3%)
	Locations with largest deviation in 2023	Japan: Fukuoka	Japan: Fukuoka
	Number of subnational locations with increasing deviation from frontiers *n* (%)	384 (98.7%)	383 (98.75%)
	Locations with largest decrease in deviation (1990–2023)	Pakistan: Balochistan	Pakistan: Islamabad Capital Territory
	Locations with largest increase in deviation (1990–2023)	Japan: Fukuoka	Japan: Fukuoka

DALYs, Disability-Adjusted Life Years. Rates are reported per 100,000 population. “Frontier” refers to optimal achievable burden based on SDI by theory.

Japanese prefectures accounted for the largest 2023 deviations. Aomori had the largest deviation for age-standardized incidence, with an effective difference of 228.5, whereas Fukuoka had the largest deviation for incidence among children and adolescents, age-standardized DALYs, and DALYs among children and adolescents, with effective differences of 116.4, 252.0, and 488.9, respectively (Table 2). Aichi showed the largest increase in deviation for incidence among children and adolescents from 1990 to 2023, with effective differences of 114.0.

Supplementary frontier plots showed a broad concentration of high DALY deviations among Japanese prefectures. In the United States, Pennsylvania was the labeled 2023 subnational outlier for age-standardized DALYs and incidence, with effective differences of 160.3 and 24.8, respectively (Supplementary Figures S2, S3).

The largest reductions in subnational deviation occurred in Pakistan and Nigeria. In Pakistan, Sindh narrowed the most in deviation in age-standardized incidence; Balochistan in age-standardized DALYs deviation; Islamabad Capital Territory in DALYs deviation among children and adolescents (Table 2). In Nigeria, Akwa Ibom showed the largest decrease in deviation for incidence among children and adolescents. Most available subnational units in Pakistan and Nigeria were classified as attaining the relevant 2023 frontiers, although deviation increased over time in many units.

In Ethiopia and Kenya, available subnational units attained the 2023 age-standardized DALYs and incidence frontiers, but deviations increased over time. As in inequality analysis for children and adolescents, Ethiopia and Kenya showed negative absolute gradients in DALYs rates in 2023, with SII values of −18.58 (−25.33 to −11.83) and −9.09 (−11.62 to −3.55), respectively (Supplementary Figure S4). However, Nigeria showed a different pattern. The SII for children and adolescents’ DALYs increased from 8.31 (2.11 to 14.50) in 1990 to 18.38 (13.59 to 23.17) in 2023, and the CI increased from 0.04 to 0.13.

## 4 Discussion

### 4.1 Frontier deviation and socioeconomic inequality in relation to ASD service organization and child safety

This study mapped geographic and temporal variation in ASD frontier deviation and socioeconomic inequality, with particular attention to children and adolescents. National deviations remained widespread, especially for DALYs among individuals younger than 20 years. Several of the largest deviations occurred in high-SDI settings, whereas pronounced absolute inequalities in some lower-SDI settings indicated that the modeled burden remained concentrated among socioeconomically disadvantaged populations. These patterns identify locations that warrant closer investigation. They should not, however, be interpreted as direct measures of healthcare system. Frontier deviation represents the distance between a modeled incidence or DALYs rate and the lowest rate observed at a comparable level of development. It does not identify the mechanism that produced that distance.

The 2023 results showed that frontier gaps remained widespread at the national level, especially for DALYs rates among children and adolescents. In 2023, 98 countries and territories fell behind the age-standardized incidence frontier and 78 fell behind the age-standardized DALYs frontier. Among the children and adolescents, 100 countries and territories lagged on incidence and 123 on DALYs. The largest deviations sat in high-income regions such as North America, Western Europe, and high-income Asia-Pacific. High SDI countries generally benefit from solid health infrastructure and a far greater supply of mental health professionals, usually backed by stronger government support, comprehensive policies and mature data systems. According to the World Health Organization (WHO) Mental Health Atlas 2020 estimated that the median mental health workforce density in high-income countries was approximately 62.2 per 100,000 population, with a median about 19.9 per 100,000 devoted to child and adolescent mental health (24). However, adequate support for children and adolescents with ASD rests on more than headcount. It requires development assessment, caregiver training, ASD-informed emergency and transport planning, with the coordination across healthcare, education, and community services (2, 9, 25, 26). Many high SDI settings still hit service bottlenecks, such as long diagnostic wait times, uneven distribution of specialists, and uneven post-diagnostic coverage, which may disproportionately affect marginalized groups, such as children from rural families (27).

The global patterns show widening inequities, most acute in low-middle and low SDI regions, where limited health resources and socioeconomic disadvantages heavily influence on both diagnosis and management of ASD (28, 29). The inequalities in ASD outcomes across sociodemographic groups indicate that targeted interventions addressing diagnostic delays, resource allocation, and healthcare infrastructure improvements are urgently required. Broader international collaboration, greater technical and financial assistance, and public health messaging that is sensitive to local context could each help close these gaps and lift ASD outcomes worldwide (30, 31).

The contrast between incidence and DALY deviation remains one of the most informative findings (32). Incidence responds strongly to diagnostic practice, awareness, referral, and data collection, whereas DALYs reflect modeled disability burden. A location may therefore appear closer to the incidence frontier but remain distant from the DALYs frontier, or vice versa. This discordance can generate hypotheses about diagnostic pathways and post-diagnostic support. A child who has been identified is not yet a child who is protected, not until recognition becomes caregiver training, safe medication storage, and guarding against wandering and elopement (33). It may indicate that identification and disability burden are changing through different processes. However, it cannot establish that recognition has, or has not, translated into effective care.

### 4.2 National trajectories require cautious interpretation of diagnostic and surveillance change

The national frontier results singled out Sweden and Japan as contrasting national level signals. Sweden narrowed its deviation more than any location from 1990 to 2023 across every national metric. Japan widened its deviation across all incidence and DALYs metrics, and in 2023 it had the largest deviation from the frontier for age-standardized incidence rate, age-standardized DALYs rate, and DALYs rate for children and adolescents. Brunei Darussalam had the largest deviation for children and adolescent’s incidence rate in 2023, but this was a metric-specific finding rather than a consistent pattern across DALYs and age-standardized outcomes.

Sweden’s frontier deviation narrowed more than any other countries and territories across both DALYs and incidence rates from 1990 to 2023, for age-standardized group and those under-20 group alike. Because the improvement was uniform rather than confined to a single metric, Sweden stands out as a benchmark for asking what drove the change (34). Several features of the Swedish system are worth considering, such as universal child health services, structured ASD surveillance, multidisciplinary assessment pathways, and rights-based disability support (34–36). Swedish child health services have been reported to reach nearly all young children, with ASD screening built into routine preschool surveillance and referral to local multidisciplinary teams (37).

Japan’s high recorded deviations may reflect multiple processes. Published studies describe increasing ASD diagnosis, delays in access to mental health care, indirect referral pathways, and gaps in ASD services (2, 38–41). Successive rounds of regulation have not visibly shifted the picture, though more recent effects may prove different. Since 2021, Japan government has expanded support for children with developmental disabilities, including ASD, through school support guidance, medical reimbursement reforms, and cross-sector data linkage, covering school transition, child and adolescent mental health care, and early identification and referral (42–44). The frontier results do not show whether these initiatives were effective. They identify a pattern that should be validated against prefectural data on age at diagnosis, waiting times, provider distribution, service use, and functional outcomes.

The near-universal increase in deviation over time also requires caution. Expanded diagnostic capacity and changes in diagnostic criteria can raise recorded incidence and modeled DALYs even when access to recognition improves. Conversely, low recorded rates can reflect under-identification rather than lower underlying need. We therefore interpret temporal change as a change in recorded deviation, not as a direct measure of worsening or improving service provision.

Brunei Darussalam is the under-20 incidence outlier. It had the largest deviation for incidence rate among children and adolescents in 2023, yet it was not the outlier for age-standardized incidence rate or DALYs rate. Brunei is a metric-specific signal, rather than as a central driver of our findings. This under-20 incidence deviation could stem from shifting diagnostic visibility, the sensitivity of a small population, or limitations in local surveillance data. Earlier work from Brunei described thin autism data infrastructure and limited service available, including the absence of a central register and constrained access for families of children with ASD (45).

The broader policy message is that national progress should be evaluated through both frontier and inequality findings. Sweden shows that sustained improvement is achievable, Japan that high development does not guarantee frontier attainment, and Brunei that child incidence can reveal specific detection signals. The inequality results then determine whether these national patterns are equitable. For child safety promotion, the aim is not simply to lower national average deviation, but to ensure that early recognition, caregiver guidance, school support, and practical safety planning reach children across socioeconomic and geographic groups.

### 4.3 Subnational frontier and inequality patterns reveal where ASD burden concentrates among disadvantaged children

The subnational findings show that national averages can conceal important within-country differences. In 2023, only 1.2 and 1.5% of subnational locations fell behind the frontier for age-standardized incidence and children and adolescents’ incidence, respectively. In contrast, 12.3% fell behind the frontier for both age-standardized DALYs and children and adolescents DALYs. Increasing deviation from the frontier was also common across subnational analyses, especially for DALYs. This indicates that many locations could appear to have attained the 2023 frontier while still drifting farther from frontier over time.

Japan dominated the subnational frontier pattern, through specific prefectures. Aomori had the largest age-standardized incidence deviation in 2023 and the steepest increase in that deviation since 1990. Fukuoka had the largest deviation in 2023 for children and adolescents’ incidence, age-standardized DALYs, and children and adolescents’ DALYs, and it also showed the largest increase in both age-standardized and children and adolescents’ DALYs from 1990 to 2023. Aichi had the largest increase in children and adolescents’ incidence deviation over the same time period. From the inequality analysis, these prefectural outliers may reflect not only high recorded burden, but also uneven distribution of specialist services, local differences in early screening, variation in school support, and unequal post-diagnostic pathways (38).

The improvement signals in Pakistan and Nigeria should also be interpreted through inequality. Balochistan (Pakistan) showed the largest fall in age-standardized DALYs deviation, and Islamabad Capital Territory showed the largest decrease in children and adolescents’ DALY deviation, and Akwa Ibom (Nigeria) showed the largest decrease in children and adolescents’ incidence deviation. All three suggest potential local progress, but the inequality implications differ. Improvements in Islamabad Capital Territory may simply reflect better service concentration in an advantaged urban administrative area, whereas improvement in Balochistan would carry far more meaningful if they mark real progress in a historically underserved setting. Akwa Ibom’s signal may indicate improved child detection or reporting, but it should be validated against local data on service access, caregiver support, and diagnostic capacity (46). These cases show why frontier improvement alone is insufficient; without inequality analysis, improvement could mean equitable expansion or concentration of services in more visible populations. Encouragingly, evidence from Pakistan on technology-assisted caregiver training and task-sharing shows that non-specialist and community-based approaches can improve support for children with developmental disorders while easing caregiver stress (47, 48).

Subnational analyses in lower SDI settings reinforce this caution. In Ethiopia and Kenya, children and adolescents’ DALYs inequality showed negative absolute gradients in 2023, indicating that child and adolescents burden fell most heavily on more disadvantaged subnational populations. That matters directly for child safety, because disadvantaged children with ASD are more likely to face delayed recognition, limited school support, fewer trained providers, and lower caregiver access to practical safety advice (49, 50). Across Ethiopia and the broader low SDI region, consistently negative SII values of relatively large magnitude. These values indicate a substantial concentration of ASD burden among socioeconomically disadvantaged populations, emphasizing pronounced health inequalities at both national and regional levels. Several factors contribute to this disparity, including delayed and uneven development of diagnostic services, demographic challenges intensified by poverty, inadequate policy responses, delayed or insufficient international aid and competing health priorities (24, 51). International aid remains critical, and urgent global collaboration is required to scale up financial and technical support, fostering the expansion of essential diagnostic and care capacities in resource-limited settings (52). Kenya’s recent nationwide ASD mapping and registration program, in order to build a database of individuals with ASD and connect them to care (53). In contrast, Nigerian children and adolescents’ DALYs rate SII and CI increased from 1990 to 2023, suggesting a growing concentration of burden toward more advantaged subnational populations, more likely a sign of stronger case recognition and service contact in developed areas than of absent need elsewhere.

Child injury prevention and safety promotion require surveillance that identify not only where burden is high, but also where vulnerable children are missed. For ASD, the most vulnerable children are often those left undiagnosed, diagnosed late, or diagnosed without receiving practical family and school support behind it. Inequality mapping is what brings them into view. Positive gradients in high SDI settings may mark recorded burden pooling where diagnostic visibility is better, while negative gradients in lower SDI settings may indicate concentration of unmet burden among disadvantaged populations.

### 4.4 Strengths and limitations

This study has several strengths. First, it covers global-to-local locations worldwide, allowing service gaps to be identified at a level more actionable than only global averages. It also evaluates incidence and DALYs rates separately and emphasizes children and adolescents. Second, the combination of frontier benchmarking and inequality analysis distinguishes two different policy problems: inefficient translation of resources into timely ASD support in some high SDI settings, and diagnostic invisibility or socioeconomic concentration of need in lower SDI settings. Third, by interpreting ASD-related service gaps through a child safety lens, the analysis connects developmental disability surveillance with practical safety promotion.

Several limitations should be considered. Firstly, this study did not directly estimate injury incidence, injury mortality, or injury-specific risk among children with ASD. The findings should therefore be interpreted as indicators of ASD-related diagnostic visibility and service readiness relevant to child safety promotion, not as direct estimates of injury burden. Secondly, this ecological study cannot establish causal relationships between frontier deviation and healthcare quality, service access, policy, or injury outcomes. Thirdly, limited availability and reliability of detailed subnational data in many low SDI countries restrict the generalizability of findings to these contexts, likely exacerbating underestimation of disparities and service gaps. Fourth, SDI does not capture urban form, intra-urban inequality, provider distribution, or school and community services. The subnational analysis did not model spatial dependence and is susceptible to differences in administrative scale and the modifiable areal unit problem.

## 5 Conclusion

Using GBD 1990–2023 estimates, this study identified substantial variation in ASD frontier deviation and socioeconomic inequality across 798 locations. Sweden showed the largest reductions in frontier deviation across all age-standardized and children and adolescents’ metrics, whereas Japan showed the largest increases and remained the main high-deviation country for age-standardized incidence, age-standardized DALYs, and children and adolescents’ DALYs. Brunei Darussalam emerged as a metric-specific outlier for children and adolescents’ incidence. Selected Japanese prefectures were prominent subnational outliers, while locations in Pakistan and Nigeria showed the largest reductions. Inequality analyses further indicated that recorded child and adolescent ASD burden is distributed unevenly across development strata. These findings provide descriptive benchmarks for ASD surveillance, early recognition, service organization, and equitable healthcare planning. Future analyses should integrate local diagnostic and service data, spatial accessibility measures, and ASD-specific safety outcomes.

## Data Availability

The datasets supporting this analysis are publicly accessible through the Global Health Data Exchange (http://ghdx.healthdata.org). Additional data can be requested from the Institute for Health Metrics and Evaluation (IHME).

## Footnotes

1
http://ghdx.healthdata.org/
