# Supplementary material for: Autism spectrum disorder burden across 798 locations, 1990–2023: frontier and inequality mapping in the context of child injury prevention and safety promotion
Source: Front Public Health. 2026 Sep 9;14:1930391. doi: 10.3389/fpubh.2026.1930391 (PMC13597694; doi:10.3389/fpubh.2026.1930391)
Supplement: Supplementary file 1 [file Supplementary_file_1.docx]

**Autism Spectrum Disorder Burden across 798 Locations, 1990–2023: Frontier and Inequality Mapping in the Context of Child Injury Prevention and Safety Promotion**

**Contents**

[Figure S1 global and regional inequality analysis of asd <20 years incidence rate: slope index of inequality and concentration index, 1990-2023 and 2023	2](#_Toc234319634)

[Figure S2 global and regional inequality analysis of asd all ages incidence rate: slope index of inequality and concentration index, 1990-2023 and 2023	4](#_Toc234319635)

[Figure S3 subnational deviations from the asd dalys frontier, 1990–2023: national and subnational trends and 2023 status across 798 locations within 14 countries	6](#_Toc234319636)

[Figure S4 subnational deviations from the asd incidence frontier, 1990–2023: national and subnational trends and 2023 status across 798 locations within 14 countries	10](#_Toc234319637)

[Figure S5 national and subnational inequality in asd < 20 years dalys rate: slope and concentration indices from 1990 to 2023	14](#_Toc234319638)

[Figure S6 national and subnational inequality in asd all ages dalys rate: slope and concentration indices from 1990 to 2023	18](#_Toc234319639)

[Figure S7 national and subnational inequality in asd all ages incidence rate: slope and concentration indices from 1990 to 2023	22](#_Toc234319640)

[Figure S8 geographic distribution of subnational deviations from the frontier in age-standardized dalys rates for asd in 2023	26](#_Toc234319641)

[Figure S9 geographic distribution of subnational deviations from the frontier in age-standardized incidence rates for asd in 2023	28](#_Toc234319642)

[Figure S10 geographic distribution of subnational deviations from the frontier for asd < 20 years dalys rates in 2023	30](#_Toc234319643)

[Figure S11 geographic distribution of subnational deviations from the frontier in age-standardized incidence rates for asd in 2023	31](#_Toc234319644)

[Figure S12 national and subnational inequality in asd < 20 years incidence rate: slope and concentration indices from 1990 to 2023	32](#_Toc234319645)

# **Figure S1 Global** and **regional inequality analysis of ASD <20 years incidence rate: slope index of inequality and concentration index, 1990-2023 and 2023**

This figure illustrates trends in both absolute and relative inequality in < 20 years autism spectrum disorder (ASD) incidence across countries from 1990 to 2023. The top-left plot shows the global slope index of inequality in incidence, with countries ranked by Socio-demographic Index (SDI), comparing 1990 and 2023. The top-middle panel shows 2023 slope indices stratified by SDI region, with fitted lines highlighting within-region gradients. The top-right bar chart presents slope index estimates and 95% uncertainty intervals for each SDI region. The bottom-left panel presents global concentration curves for 1990 and 2023, with the corresponding concentration index (CI) values summarizing relative inequality. The bottom-middle plot shows 2023 concentration curves by SDI region, and the bottom-right chart displays the concentration index and uncertainty intervals for each region.

# **Figure S2 Global** and **regional inequality analysis of ASD all ages incidence rate: slope index of inequality and concentration index, 1990-2023 and 2023**

This figure illustrates trends in both absolute and relative inequality in autism spectrum disorder (ASD) incidence across countries from 1990 to 2023. The top-left plot shows the global slope index of inequality in incidence, with countries ranked by Socio-demographic Index (SDI), comparing 1990 and 2023. The top-middle panel shows 2023 slope indices stratified by SDI region, with fitted lines highlighting within-region gradients. The top-right bar chart presents slope index estimates and 95% uncertainty intervals for each SDI region. The bottom-left panel presents global concentration curves for 1990 and 2023, with the corresponding concentration index (CI) values summarizing relative inequality. The bottom-middle plot shows 2023 concentration curves by SDI region, and the bottom-right chart displays the concentration index and uncertainty intervals for each region.

# Figure S3 Subnational deviations from the ASD DALYs frontier, 1990–2023: national and subnational trends and 2023 status across 798 locations within 14 countries

Each row presents a country-specific analysis of age-standardized disability-adjusted life years (DALYs) rates attributable to autism spectrum disorder (ASD) at the national and subnational levels from 1990 to 2023 (left panel), and the deviation from the estimated frontier in 2023 (right panel). The left panels show temporal trends in DALYs rates against the Socio-demographic Index (SDI). The black curve representing the achievable frontier. The right panels depict each subnational unit’s position relative to the 2023 frontier, identifying those that attained or fell behind the frontier and indicating the direction of change since 1990. Countries included are: Japan, the United States, Norway, the United Kingdom, Mexico, Brazil, Iran, Pakistan, Indonesia, Ethiopia, Kenya, South Africa, Nigeria, and India. Marked subnational units illustrate specific examples significant deviation from the frontier.

# Figure S4 Subnational deviations from the ASD incidence frontier, 1990–2023: national and subnational trends and 2023 status across 798 locations within 14 countries

Each pair of panels illustrates trends in age-standardized incidence rates of autism spectrum disorder (ASD) at the national and subnational levels between 1990 and 2023 (left), and the deviation from the frontier in 2023 (right). Left panels display ASD incidence rates plotted against Socio-demographic Index (SDI). The black curve representing the achievable frontier. Points are colored by year to show longitudinal trajectories. Right panels show each subnational location’s distance from the 2023 frontier, indicating whether it attained or fell behind the optimal benchmark and whether its deviation increased or decreased since 1990. Countries included are: Japan, the United States, Norway, the United Kingdom, Mexico, Brazil, Iran, Pakistan, Indonesia, Ethiopia, Kenya, South Africa, Nigeria, and India. Notable subnational areas are annotated for emphasis.

# Figure S5 National and subnational inequality in ASD < 20 years DALYs rate: slope and concentration indices from 1990 to 2023

This figure presents changes in absolute and relative inequality in autism spectrum disorder (ASD) < 20 years DALYs rates from 1990 to 2023 using slope index of inequality (SII; left panels) and concentration index (CI; right panels). The SII panels plot ASD DALYs rates by relative rank of Socio-demographic Index (SDI). Solid lines represent the fitted regression lines for 1990 (orange) and 2023 (green), while the shaded areas denote the 95% uncertainty intervals around these slopes. The CI panels display concentration curves showing the cumulative share of ASD DALYs rate ranked by population-weighted SDI or GDPPC, with CI values summarizing relative inequality. Countries included are: Japan, the United States, Norway, the United Kingdom, Mexico, Brazil, Iran, Pakistan, Indonesia, Ethiopia, Kenya, South Africa, and Nigeria. Negative SII and CI values indicate higher ASD DALYs concentrated among more disadvantaged populations, whereas positive values reflect higher DALYs among more advantaged populations.

# Figure S6 National and subnational inequality in ASD all ages DALYs rate: slope and concentration indices from 1990 to 2023

This figure presents changes in absolute and relative inequality in autism spectrum disorder (ASD) all ages DALYs rates from 1990 to 2023 using slope index of inequality (SII; left panels) and concentration index (CI; right panels). The SII panels plot ASD DALYs rates by relative rank of Socio-demographic Index (SDI). Solid lines represent the fitted regression lines for 1990 (orange) and 2023 (green), while the shaded areas denote the 95% uncertainty intervals around these slopes. The CI panels display concentration curves showing the cumulative share of ASD DALYs rate ranked by population-weighted SDI or GDPPC, with CI values summarizing relative inequality. Countries included are: Japan, the United States, Norway, the United Kingdom, Mexico, Brazil, Iran, Pakistan, Indonesia, Ethiopia, Kenya, South Africa, and Nigeria. Negative SII and CI values indicate higher ASD DALYs concentrated among more disadvantaged populations, whereas positive values reflect higher DALYs among more advantaged populations.

# Figure S7 National and subnational inequality in ASD all ages incidence rate: slope and concentration indices from 1990 to 2023

This figure presents changes in absolute and relative inequality in autism spectrum disorder (ASD) all ages incidence ratesfrom 1990 to 2023 using slope index of inequality (SII; left panels) and concentration index (CI; right panels). The SII panels plot ASD incidence rates by relative rank of Socio-demographic Index (SDI). Solid lines represent the fitted regression lines for 1990 (orange) and 2023 (green), while the shaded areas denote the 95% uncertainty intervals around these slopes. The CI panels display concentration curves showing the cumulative share of ASD incidence rate ranked by population-weighted SDI or GDPPC, with CI values summarizing relative inequality. Countries included are: Japan, the United States, Norway, the United Kingdom, Mexico, Brazil, Iran, Pakistan, Indonesia, Ethiopia, Kenya, South Africa, and Nigeria. Negative SII and CI values indicate higher ASD incidence concentrated among more disadvantaged populations, whereas positive values reflect higher incidence among more advantaged populations.

# Figure S8 Geographic distribution of subnational deviations from the frontier in age-standardized DALYs rates for ASD in 2023

This figure presents the subnational deviation from the estimated frontier in age-standardized disability-adjusted life years (DALYs) rates attributable to autism spectrum disorder (ASD) in 2023 across 14 countries. Each map shows the magnitude of deviation (per 100,000 population) between observed and frontier DALYs rates for each subnational unit. Higher values indicate a greater distance between the observed rate and the estimated frontier. The countries included are Japan, the United States, Norway, the United Kingdom, Mexico, Brazil, Iran, Pakistan, Indonesia, Ethiopia, Kenya, South Africa, Nigeria, and India. Color gradients reflect the distribution of deviation within each country, with NA indicating missing data for certain locations.

# Figure S9 Geographic distribution of subnational deviations from the frontier in age-standardized incidence rates for ASD in 2023

This figure presents the subnational deviation from the estimated frontier in age-standardized incidence rates attributable to autism spectrum disorder (ASD) in 2023 across 14 countries. Each map shows the magnitude of deviation (per 100,000 population) between observed and frontier incidence rates for each subnational unit. Higher values indicate a greater distance between the observed rate and the estimated frontier. The countries included are Japan, the United States, Norway, the United Kingdom, Mexico, Brazil, Iran, Pakistan, Indonesia, Ethiopia, Kenya, South Africa, Nigeria, and India. Color gradients reflect the distribution of deviation within each country, with NA indicating missing data for certain locations.

# Figure S10 Geographic distribution of subnational deviations from the frontier for ASD < 20 years DALYs rates in 2023

This figure presents the deviation from the estimated frontier in < 20 years disability-adjusted life years (DALYs) rates attributable to autism spectrum disorder (ASD) in 2023 across 14 countries. Countries are color-coded based on their deviation magnitude, with dark blue representing the lowest deviation, and the dark red indicating the highest deviations.

# Figure S11 Geographic distribution of subnational deviations from the frontier incidence rates for children and adolescents with ASD in 2023

This figure presents the deviation from the estimated frontier in < 20 years incidence rates attributable to autism spectrum disorder (ASD) in 2023 across 14 countries. Countries are color-coded based on their deviation magnitude, with dark blue representing the lowest deviation, and the dark red indicating the highest deviations.

# Figure S12 National and subnational inequality in ASD < 20 years incidence rate: slope and concentration indices from 1990 to 2023

This figure presents changes in absolute and relative inequality in autism spectrum disorder (ASD) < 20 years incidence rates from 1990 to 2023 using slope index of inequality (SII; left panels) and concentration index (CI; right panels). The SII panels plot ASD incidence rates by relative rank of Socio-demographic Index (SDI). Solid lines represent the fitted regression lines for 1990 (orange) and 2023 (green), while the shaded areas denote the 95% uncertainty intervals around these slopes. The CI panels display concentration curves showing the cumulative share of ASD incidence rate ranked by population-weighted SDI or GDPPC, with CI values summarizing relative inequality. Countries included are: Japan, the United States, Norway, the United Kingdom, Mexico, Brazil, Iran, Pakistan, Indonesia, Ethiopia, Kenya, South Africa, and Nigeria. Negative SII and CI values indicate higher ASD incidence concentrated among more disadvantaged populations, whereas positive values reflect higher incidence among more advantaged populations.
